# Efficacy and safety of autologous platelet-rich plasma for diabetic foot ulcer healing: a systematic review and meta-analysis of randomized controlled trials

**DOI:** 10.1186/s13018-023-03854-x

**Published:** 2023-05-19

**Authors:** Juan Deng, Mei Yang, Xingyu Zhang, Hongmin Zhang

**Affiliations:** Department of Endocrinology, People’s Hospital of Chongqing Liang Jiang New Area, Chongqing, 401121 China

**Keywords:** Platelet-rich plasma, Diabetic foot ulcer, Randomized controlled trials, Meta-analysis

## Abstract

**Background:**

The occurrence of a diabetic foot ulcer (DFU) is a significant complication of diabetes that often precedes the need for amputation. Autologous platelet-rich plasma (Au-PRP), a substance abundant in various growth factors and cytokines, is increasingly being recognized as a promising method for promoting ulcer healing due to its potential similarities to the physiological wound healing process.

**Methods:**

The databases Medline, EMBASE, PubMed, and the Cochrane Library were systematically accessed on January 26, 2023, without any consideration for the date of publication. The selection and assessment of research studies were conducted autonomously, based on predetermined criteria and methodological standards. Two researchers gathered data and evaluated the potential for bias separately. We utilize the Stata 17.0 software to conduct data analysis and generate relevant visual representations.

**Results:**

The results of the meta-analysis indicate that autologous PRP has a significant positive effect on the healing rate (RR = 1.42, 95% CI 1.30–1.56, *P* < 0.001), reduces the healing time (MD = − 3.13, 95% CI − 5.86 to − 0.39, *P* < 0.001), accelerates the reduction of ulcer area (MD = 1.02, 95% CI 0.51–1.53, *P* < 0.001), decreases the rate of amputation (RR = 0.35, 95% CI 0.15–0.83, *P* < 0.001), and does not increase the incidence of adverse events (RR = 0.96, 95% CI 0.57–1.61, *P* > 0.05) when compared to conventional therapy.

**Conclusions:**

Au-PRP therapy has been shown to facilitate the process of wound healing and represents a viable and secure therapeutic alternative for individuals with DFU.

**Supplementary Information:**

The online version contains supplementary material available at 10.1186/s13018-023-03854-x.

## Introduction

Diabetes mellitus (DM) is a chronic metabolic disease characterized by hyperglycemia [1]. As per the findings of the research, the global prevalence of DM was estimated to be approximately 436 million individuals in the year 2019. It is projected that the number of individuals affected by DM worldwide will escalate to 700 million by the year 2045 [2]. The incidence of DM and its associated complications has not only significantly diminished patients' quality of life, but also posed a substantial threat to their survival, thereby presenting significant economic and healthcare obstacles. Diabetic foot ulcer (DFU) is a prevalent complication of DM. Research indicates that the global annual incidence of DFU is approximately 6.3% [3]. DFU can be attributed to several factors such as inadequate management of blood glucose levels, structural abnormalities of the foot, neurological impairments, compromised circulation, and physical injury [4, 5]. Once the DFU is formed, it is easy to develop into a chronic refractory wound, which eventually leads to amputation or even death. In addition, due to the persistence of ulcer formation factors, even if the wound heals successfully, ulcers are still easy to recur in a short period of time [4]. The cost of DFU in the USA increased fivefold from 2005 to 2010, spending more than $1 billion a year on DFU care [6, 7]. As of 2017, the expenditure for prevention and treatment of DM amounted to approximately $237 billion, with a significant portion of almost 33% allocated towards DFU. This proportion is comparable to the expenses incurred for prevalent types of cancer [8]. The efficacy of traditional therapeutic interventions, including glycemic control, neural nourishment, anti-infective measures, localized decompression, comprehensive debridement and dressing modifications, sufficient drainage, enhanced microcirculation, and vascular restructuring, is suboptimal in facilitating the healing of diabetic foot ulcers [9–11].

PRP is a concentrated plasma preparation that contains a high concentration of platelets. It is derived from either autologous or allogeneic whole blood of patients [12]. Based on the origin of the blood, platelet-rich plasma (PRP) can be classified into two categories: autologous PRP (Au-PRP) and allogeneic PRP (Al-PRP). Due to its autologous nature, Au-PRP is not susceptible to immune rejection, thus making it the predominant form of PRP utilized in clinical settings. The therapeutic mechanism of Au-PRP in the treatment of DFU is attributed to its rich composition of growth factors, white blood cells, antimicrobial peptides, fibrin, and diverse cytokines. These constituents work in tandem to regulate the inflammatory response, expedite the formation of extracellular matrix, promote angiogenesis, and facilitate re-epithelialization, ultimately leading to the healing of the ulcer. The utilization of Au-PRP as a potential treatment for DFU may prove advantageous based on the pathophysiological mechanisms of wound healing in diabetes. Nevertheless, the current body of evidence is insufficient to substantiate this hypothesis [13]. The utilization of Au-PRP has been suggested as a potential treatment option for DFU that have failed to heal despite standard therapy [14].

The objective of this study is to investigate, assess, and synthesize scientific data pertaining to the safety and therapeutic effectiveness of Au-PRP in the management of DFU in comparison with conventional treatment or any other substitute therapy.

## Materials and methods

During the systematic review process and subsequent reporting of our results, we maintained adherence to the Preferred Reporting Items for Systematic Reviews and Meta-Analyses (PRISMA) guidelines [15]. Since the information utilized in this article was sourced from published materials, there was no need for informed consent or ethical approval. Two researchers conducted a systematic search of pertinent studies, independently determined their eligibility, extracted data, and evaluated the quality of the research. The two researchers were required to reach a consensus and resolve any points of disagreement.

### Search strategy

The electronic databases of Medline, EMBASE, PubMed, and the Cochrane Library were searched on January 26, 2023. The vocabulary and grammar were adjusted in accordance with the database through specific modifications. The study utilized the search phrases "platelet-rich plasma" in conjunction with "foot ulcer" or "diabetic foot." There were no restrictions on language or timeframe. The PubMed search strategy is shown below: (("Platelet Rich Plasma"[MeSH Terms] OR "Plasma, Platelet-Rich"[MeSH Terms] OR "Platelet-rich Plasma Gel"[MeSH Terms] OR "PRP"[MeSH Terms] OR "Platelet-Rich Plasma"[MeSH Terms])) AND ("Diabetic Foot"[MeSH Terms] OR "Foot Ulcer, Diabetic"[MeSH Terms] OR "Feet, Diabetic"[MeSH Terms] OR "Diabetic foot ulcer wounds"[MeSH Terms] OR "Diabetic Feet"[MeSH Terms] OR "Diabetic foot ulcer"[MeSH Terms] OR "Diabetic foot Wound"[MeSH Terms]) AND ("Randomized Controlled Trial"[Publication Type] OR "Randomized"[MeSH Terms] OR "Placebo"[MeSH Terms]). In order to conduct a comprehensive systematic search, the reference lists of all relevant articles were scrutinized to identify any additional studies that met the established inclusion criteria.

### Inclusion criteria and exclusion criteria

The following requirements required to be met by studies to be included in the systematic review: (1) Design: randomized controlled trials (RCTs). (2) Population aged between 18 and 65 years who have DFU. (3) Intervention: any product containing a supraphysiologic concentration of autologous platelets. (4) Comparator: conventional therapy, no intervention, and alternative treatment for foot ulcers. (5) Outcome measures: proportion of DFU that is completely healed, total epithelialized area (cm2), ulcer volume decrease (cm3), duration to complete wound healing, wound complications, adverse events, amputation rate.

The exclusion criteria were as follows: (1) repeatedly published literature; (2) studies with incomplete or unclear analytical data and inconsistent outcome indicators; (3) studies with poor quality and lack of original data.

### Data extraction

Two reviewers were required to independently scrutinize the literature and extract the pertinent data. The results obtained required a process of cross-verification, and in case of any inconsistencies, they were subject to thorough discussion and resolution. During the literature screening process, the researchers initially review the title and abstract of the articles. Subsequently, they scrutinize the complete text to ascertain its inclusion in the study, while eliminating any overtly irrelevant content. The standardized Excel files contain extracted and recorded requisite data, which includes the surname of the first author, publication year, country, study design, demographic information of participants, treatment strategy, ulcer classification, PRP preparation, and PRP application. In instances where the published report lacked pertinent data, the investigators of the original study were contacted via email to request access to the unpublished data.

### Quality assessment

The quality of the included studies was assessed by the Cochrane Collaboration’s risk of bias tool [16]. Two reviewers independently evaluated the following domains: random sequence generation, allocation concealment, blinding of participants and personnel, incomplete outcome data, selective reporting, and other potential sources of bias. Each domain was judged as having a low, unclear, or high risk of bias. Disagreements between reviewers were resolved through discussion or consultation with a third reviewer, if necessary.

### Statistical analyses

The heterogeneity between studies was assessed using Chi-square statistics and quantified by the size of I^2^. The heterogeneity of the included studies was assessed using the I^2^ statistic. I^2^ values greater than 50% indicated significant heterogeneity, whereas values of 0% signified no observable heterogeneity. When I^2^ was greater than 50%, the random effect model was chosen; when I^2^ was less than 50%, the fixed-effect model was employed. To assess the robustness of our results and identify any potential influence of individual studies on the overall effect size, we conducted a sensitivity analysis. This analysis involved sequentially removing each study from the meta-analysis and recalculating the overall effect size, examining whether the point estimates of the overall effect remained within the 95% confidence interval of the initial combined effect. The assessment of publication bias was conducted through the utilization of Egger's test and funnel plots. Statistical significance was determined by considering a two-sided P value of less than 0.05 in all analyses. The Stata version 17 (StataCorp, College Station, TX, USA) was utilized to analyze data from randomized controlled trials (RCTs) that satisfied the inclusion criteria. The certainty of the synthesized evidence was evaluated using the GRADEprofiler grading system following the GRADE (Grading of Recommendations, Assessment, Development and Evaluations) approach [17].

## Results

### Search results and study selection

**Fig. 1:**
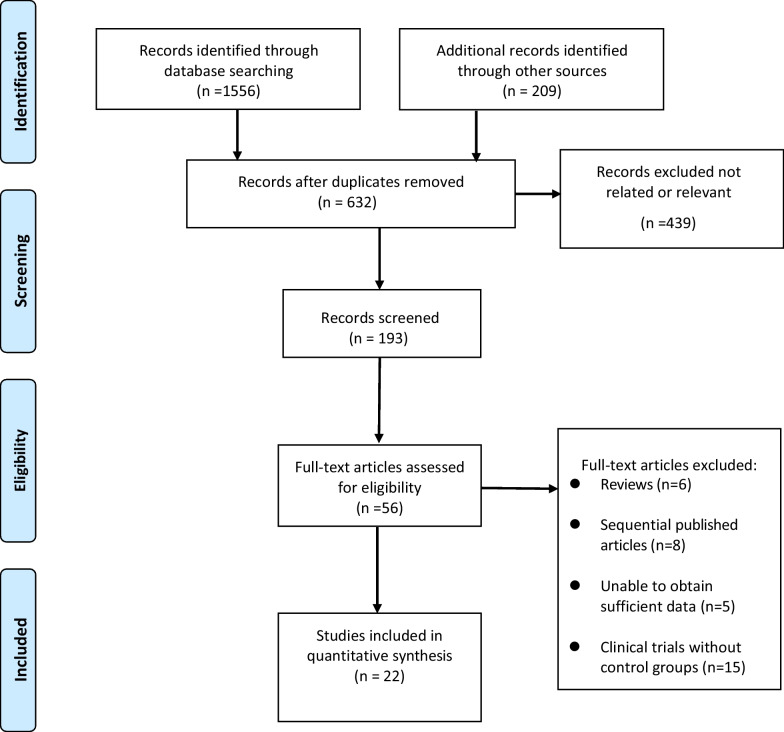
Selection process of included studies

The initial query of the electronic databases yielded a total of 1765 research studies. Following the elimination of redundant literature, careful examination of titles and abstracts, and rigorous adherence to the established inclusion and exclusion criteria, a total of 56 relevant pieces of literature were procured, while 34 were deemed unsuitable for further analysis. Ultimately, a total of 22 articles were included [18–39]. The literature screening process and results are shown in Fig. 1.

### Study characteristics

The selected trials encompassed a total of 1559 individuals who presented with wounds resulting from diabetic foot ulcers. Of these participants, 785 were subjected to treatment with platelet-rich plasma, while the remaining 774 were assigned to a control group. At the onset of the study, the quantity of individuals involved varied between 13 and 200. Table 1 presents a concise overview of the key characteristics of the studies that were incorporated. All the publications were released in the English language and spanned the years between 1992 and 2022. Each study analyzed data pertaining to a solitary ulcer per participant. Nineteen research studies were conducted to compare the effectiveness of PRP in combination with standard care versus standard care alone. One study was conducted to evaluate the efficacy of PRP in conjunction with standard care as opposed to platelet-poor plasma in conjunction with standard care [23]. One study was conducted to evaluate the efficacy of PRP in conjunction with standard care against oxidized regenerated cellulose/collagen biomaterial in conjunction with standard care [21]. Another study conducted a comparison between the administration of PRP in conjunction with standard care and the application of Saline gel in conjunction with standard care [20].

**Table 1:** Characteristics of studies included in the meta-analysis

Author	Year	Study design	Country	Total/PRP	Treatment strategy	Treatment strategy	Ulcer classification	PRP preparation	PRP application
Tofigh	2022	RCT	Iran	161/81	PRP"	Control"	Wagner classification: I, II, III, IV, V	Peripheral blood centrifuge at a rate of 2000–3200 rpm for 10–15 min	PRP gel applied on ulcer with Vaseline gauze, few layers of sterile gauze, and non-compressible bandage. This was repeated twice weekly
Meamar	2021	RCT	Iran	17/10	PRP + SC	SC	Texas classification: IA, IIA, IC, IIC	Peripheral blood centrifuge at a rate of 2000–3200 rpm for 10–15 min	PRP gel applied on ulcers after irrigation with 0.9% saline twice weekly covered with non-absorbing dressing
Helmy	2021	RCT	Egypt	80/40	PRP + SC	SC	Wagner classification: I-IV	NC	PRP gel applied on ulcers covered with two pieces of dry sterile gauzes. PRP dressing was performed only once at the beginning of study
Hossam	2021	RCT	Iran	160/80	PRP + SC	SC	Wagner classification: I, II, III, IV, V	Peripheral blood centrifuge at a rate of 2000–3200 rpm for 10–15 min	PRP gel applied on ulcers covered with two pieces of dry sterile gauzes. PRP dressing was performed only once at the beginning of study
Alamdari	2021	RCT	Iran	90/43	PRP + SC	SC	Ulcers that had exposed bone or bone, involvement were excluded	Peripheral blood centrifuge at a rate of 2000–3200 rpm for 10–15 min	PRP gel applied on ulcers covered with Vaseline gauze and then a dressing. This was repeated twice weekly
Habeeb	2020	RCT	Egypt	44/22	PRP + SC	SC	Wagner classification: I, II, III, IV, V	Peripheral blood was centrifuged at 2000 rpm A for 10 min	PRP gel applied on ulcers covered with vapor-permeable film (Tegaderm, 3 M)
Liao	2020	RCT	China	200/100	PRP + SC	SC	NC	1. Peripheral blood was centrifuged for 1.5 min. 2. It was delivered by autologel system	PRP gel applied on ulcers covered with vapor-permeable film (Tegaderm, 3 M)
Elsaid	2020	RCT	Egypt	24/12	PRP + SC	SC	Wagner classification: I-IV	1. Peripheral blood was centrifuged at 3600 rpm. 2. A second centrifugation at 2400 rpm	PRP gel applied on ulcer with Vaseline gauze, few layers of sterile gauze, and non-compressible bandage. This was repeated twice weekly
Rainys	2019	RCT	Lithuania	69/35	PRP + SC	SC	NC	Peripheral blood centrifuge at a rate of 2000–3200 rpm for 10–15 min	PRP gel applied on ulcer with contact layer dressing covered with non-absorbent foam dressing changed every 3–4 d
Singh	2018	RCT	India	55/29	PRP + SC	SC	Ulcers that had exposed bone or bone, involvement were excluded	Peripheral blood centrifuge at a rate of 2000–3200 rpm for 10–15 min	An appropriate amount of the PRP (approximately 3–4 ml of PRP for a 5 × 10 cm ulcer) was injected at various points along the wound edges once a week
Ahmed	2017	RCT	Egypt	56/28	PRP + SC	SC	Texas classification: IA, IIA, IC, IIC	1. Peripheral blood was centrifuged at 1500 rpm for 5 min. 2. A second centrifugation at 3500 rpm for 5 min	PRP gel applied on ulcers after irrigation with 0.9% saline twice weekly covered with non-absorbing dressing
Karimi	2016	RCT	Iran	50/25	PRP + SC	SC	Wagner classification: I, II	Peripheral blood was centrifuged at 2000 rpm A for 10 min	PRP gel applied on ulcers covered with two pieces of dry sterile gauzes. PRP dressing was performed only once at the beginning of study
Li	2015	RCT	China	103/48	PRP + SC	SC	Wagner classification: I, IV	1. Peripheral blood was centrifuged for 1.5 min. 2. It was delivered by autologel System	Weekly topical application of PRP gel with covered with standard dressing changed weekly
Li	2012	RCT	China	117/59	PRP + SC	SC	Wagner classification: I, II, III, IV, V	1. Peripheral blood was centrifuged at 313 × g for 4 min 2. A second centrifugation at 1252 × g for 6 min	PRP gel applied on ulcers covered with two pieces of dry sterile gauzes. PRP dressing was performed only once at the beginning of study
Saad Setta	2011	RCT	Egypt	24/12	PRP + SC	SC	Ulcers that had exposed tendons, ligaments or bone were excluded	1. Peripheral blood was centrifuged at 1007 × g. 2. A second centrifugation at 477.5 × g	PRP gel applied on ulcers covered with Vaseline gauze and then a dressing. This was repeated twice weekly
Jeong	2010	RCT	Korea	100/52	PRP + SC	PPP + SC	Texas classification: IA, IIA, IC, IIC	Peripheral blood centrifuge at a rate of 2000–3200 rpm for 10–15 min	PRP gel applied on ulcers covered with two pieces of dry sterile gauzes. PRP dressing was performed only once at the beginning of study
Friese	2007	RCT	Netherlands	42/21	PRP + SC	SC	Wagner classification: I, II, III	It was delivered by Harvest System (Harvest Technologies, Plymouth, MA)	PRP gel applied on ulcer every two weeks
Kakagia	2007	RCT	Greece	32/16	PRP + SC	SC	NC	It was delivered by Gravitational Platelet Separation System (GPS, Biomet)	PRP gel applied on ulcers covered with vapor-permeable film (Tegaderm, 3 M)
Driver	2006	RCT	USA	72/40	PRP + ORC/CB + SC	ORC/CB + SC	Texas classification: IA	1. Peripheral blood was centrifuged for 1.5 min. 2. It was delivered by autologel System(AutoloGel, Cytomedix, Gaithersburg, MD	Weekly topical application of PRP gel with covered with standard dressing changed weekly
Saldalamacchia	2004	RCT	Italy	14/7	PRP + SC	Saline gel + SC	Wagner classification: II, III	NC	Weekly topical application of PRP gel with covered with standard dressing changed weekly
Steed	1996	RCT	USA	36/18	PRP + SC	SC	Wagner classification: I, II, III	Delivered by Gravitational Platelet Separation System (GPS, Biomet)	PRP gel applied on ulcers covered with Vaseline gauze and then a dressing. This was repeated twice weekly
Steed	1992	RCT	USA	13/7	PRP + SC	SC	NC	Peripheral blood centrifuge at a rate of 2000–3200 rpm for 10–15 min	PRP gel applied on ulcer every two weeks

NC, not clear; ORC/CB, oxidized regenerated cellulose/collagen biomaterial; PPP, platelet-poor plasma; SC, standard care

### Results of quality assessment

**Fig. 2:**
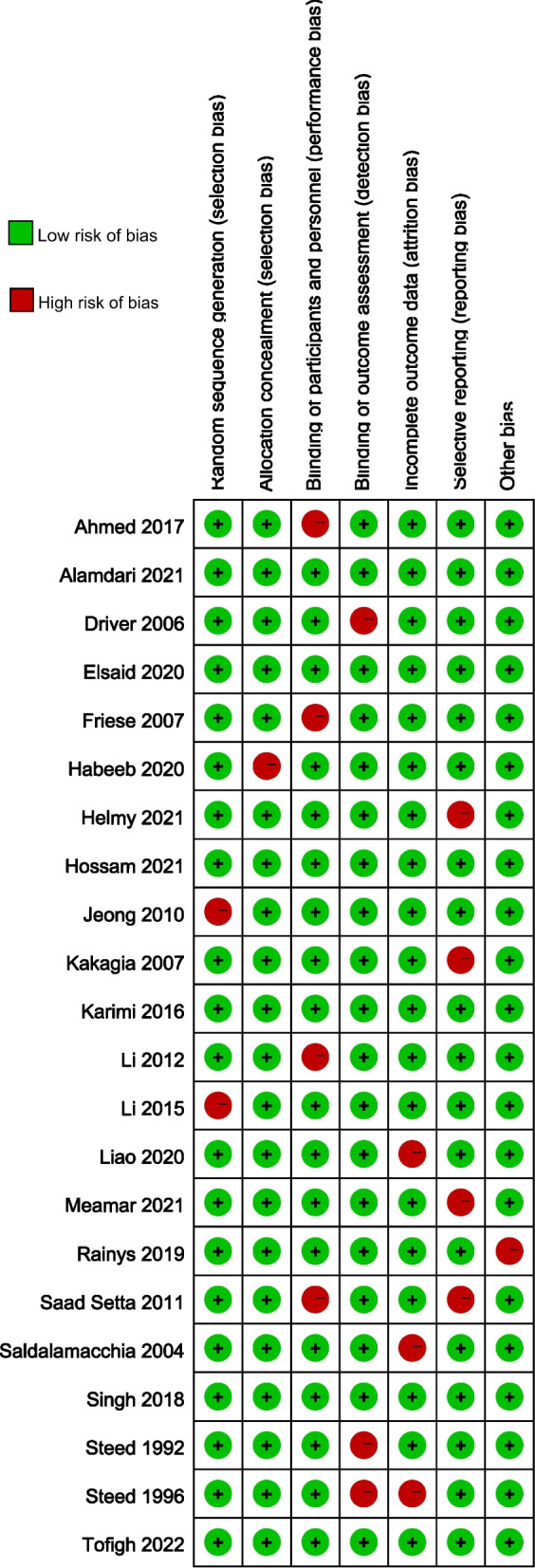
Risk of bias summary graph for the included studies

The evaluation of bias risk was conducted across multiple domains in the 22 studies that were included. Seven studies demonstrated a low risk of bias in all categories, indicating a high level of methodological rigor. However, 20% of the studies were found to have a high risk of bias in the domain of blinding of participants and personnel. This suggests that the potential for performance bias might have influenced the outcomes in these studies. Furthermore, in 21% of the included randomized controlled trials, a high risk of selective reporting bias was observed. This indicates that the possibility of incomplete or selective outcome reporting may have affected the overall results of these studies (Fig. 2).

### Overall healing rate of DFU

**Fig. 3:**
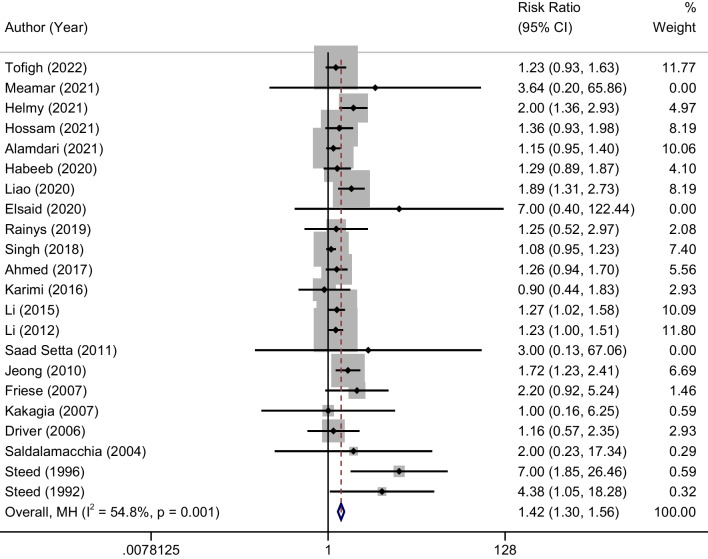
Forest plot comparing the efficacy of autologous platelet-rich plasma against control on the healing of diabetic foot ulcers

In the literature, 22 studies have documented the healing rate of Au-PRP compared to conventional treatment for DFU. The ulcer healing rate for the Au-PRP group varied between 12.5% (2/16) and 100% (29/29). The results of the control treatment group indicated that the conventional treatment did not result in any ulcer healing, as evidenced by the lowest healing rate of 0%. However, the highest healing rate of 92.3% (24/26) was observed in this group. The findings of the meta-analysis indicate that the use of Au-PRP is associated with a notable enhancement in the healing rate of DFU when compared to conventional treatment. This difference is statistically significant (RR = 1.42, 95% CI 1.30–1.56, *P* < 0.001; Fig. 3). The outcomes of the heterogeneity test (*P* < 0.001 and *I*^2^ = 54.8%) indicated the presence of heterogeneity among the studies that were incorporated in the analysis.

### Time to complete wound healing

**Fig. 4:**
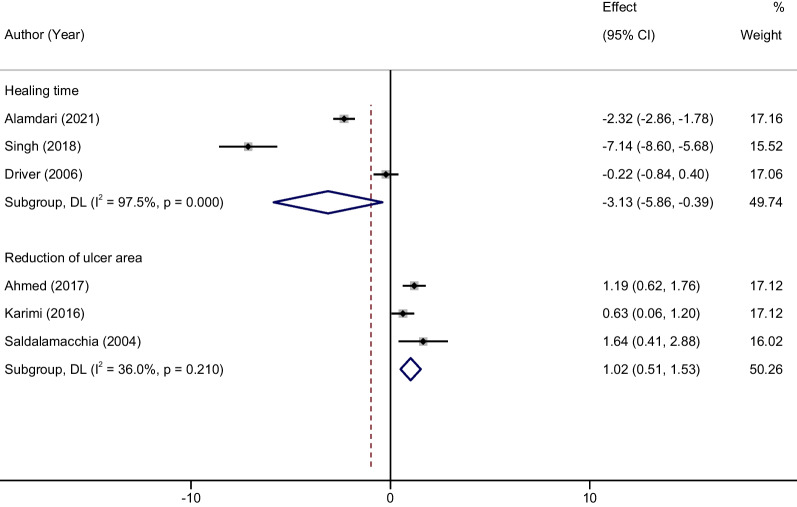
Forest plot comparing the efficacy of autologous platelet-rich plasma against control on the healing time and reduction of ulcer area of diabetic foot ulcers

A total of 3 studies [20, 32, 36] were conducted to compare the healing time of DFU between Au-PRP and conventional therapy alone. The results of meta-analysis showed that Au-PRP could significantly shorten the healing time of DFU compared with conventional therapy, and the difference was statistically significant (MD = − 3.13, 95% CI − 5.86 to − 0.39, *P* < 0.001; Fig. 4). The results of heterogeneity test (*P* < 0.0001, and *I*^2^ = 97.5%) suggested that there was some heterogeneity among the included studies.

### Ulcer volume reduction

Three studies [25, 26, 39] reported the changes of ulcer area before and after treatment in the two groups. The results of meta-analysis showed that Au-PRP could significantly accelerate the reduction of DFU area compared with conventional therapy, and the difference was statistically significant (MD = 1.02, 95% CI 0.51–1.53,* P* < 0.001; Fig. 4). The results of heterogeneity test (*P* = 0.210, and *I*^2^ = 36%) suggested that there was not heterogeneity among the included studies.

### Amputation rate

**Fig. 5:**
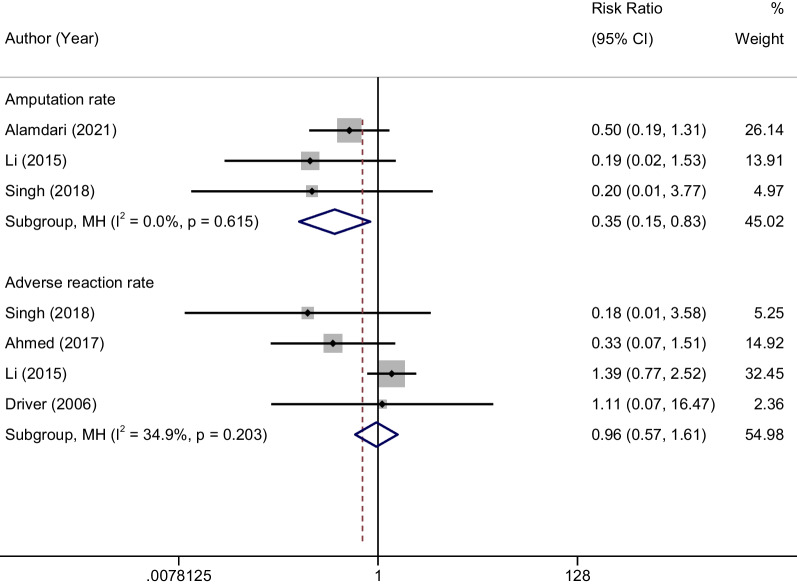
Forest plot comparing the efficacy of autologous platelet-rich plasma against control on the amputation rate and adverse reaction rate

A total of 3 studies [32, 36, 37] reported amputation rates in two groups of patients. The results of meta-analysis showed that Au-PRP could significantly reduce the rate of amputation compared with conventional therapy, and the difference was statistically significant (RR = 0.35, 95% CI 0.15–0.83, *P* < 0.001; Fig. 5). The results of heterogeneity test (*P* = 0.615, and *I*^2^ = 0.0%) suggested that there was not heterogeneity among the included studies.

### Adverse events

A total of 4 studies [20, 26, 36, 37] reported the occurrence of adverse reactions including local fever, local itching, tingling, ant sensation, local infection, dermatitis, etc. The results of meta-analysis showed that Au-PRP could not increase the incidence of adverse events compared with conventional therapy (RR = 0.96, 95% CI 0.57–1.61, *P* > 0.05; Fig. 5). The results of heterogeneity test (*P* = 0.203, and *I*^2^ = 34.9%) suggested that there was not heterogeneity among the included studies.

### Publication bias

**Fig. 6:**
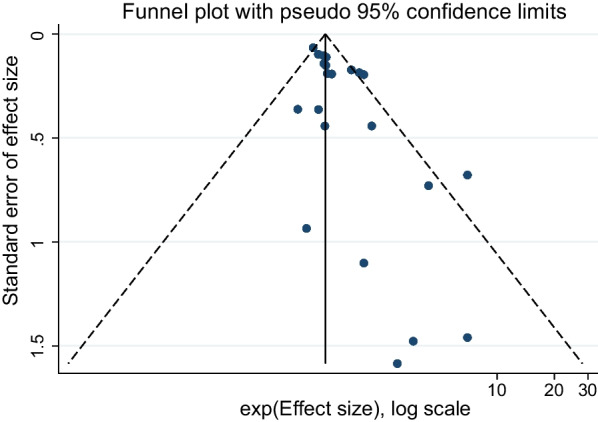
Funnel chart of the correlation between vitamin D level in children and dental caries risk

The funnel plots constructed with the observed study showed symmetry, and no significant publication bias was detected in funnel plots (Fig. 6).

### Sensitivity analysis

**Fig. 7:**
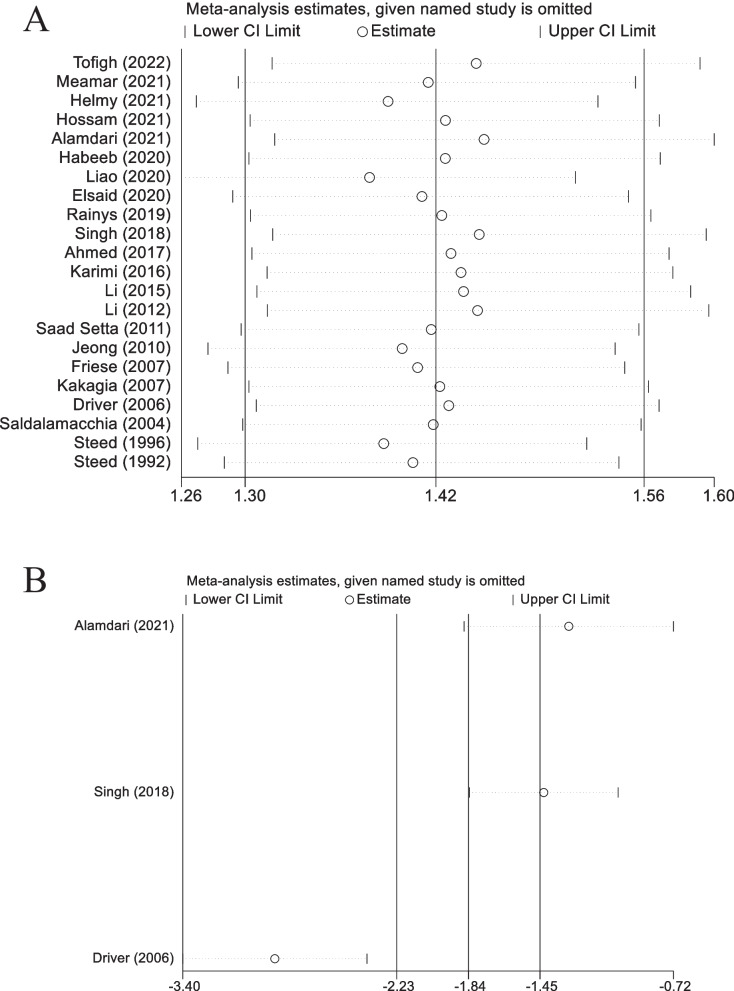
Sensitivity analysis for "overall healing rate of DFU" (**A**) and "time to complete wound healing" (**B**)

Overall healing rate of DFU: The sensitivity analysis revealed that our results were robust and not unduly influenced by any single study. After sequentially excluding each study and recalculating the overall effect size, the point estimates consistently fell within the 95% confidence interval of the initial combined effect. This finding indicates that the conclusions drawn from our meta-analysis remain stable and reliable, even when considering potential variations across individual studies (Fig. 7A).

Time to complete wound healing: Our second sensitivity analysis, which involved sequentially removing each of the three studies and recalculating the overall effect size, revealed that the point estimates consistently fell outside the 95% confidence interval of the initial combined effect. This finding suggests that there is considerable variability among the individual studies, and the overall effect size might be influenced by one or more of the included studies (Fig. 7B).

### Certainty of evidence

The certainty of the evidence for each outcome was assessed using the GRADEprofiler grading system based on the GRADE approach. The evaluation considered the risk of bias, inconsistency, indirectness, imprecision, and publication bias. The certainty of evidence for each outcome was classified as high, moderate, low, or very low. A detailed summary of the certainty of evidence assessment for each outcome is provided in Table 2.

**Table 2:** Summary of Certainty of Evidence Assessment for Each Outcome Using the PROfiler Grading System

Outcome	Certainty of Evidence	Risk of Bias	Inconsistency	Indirectness	Imprecision	Publication Bias
Overall Healing Rate of DFU	High	Low	Moderate	Low	Low	Low
Time to Complete Wound Healing	Moderate	Moderate	High	Low	Moderate	Low
Ulcer Volume Reduction	High	Low	Low	Low	Low	Low
Amputation Rate	Moderate	Low	Low	Moderate	Low	Low
Adverse Events	Low	Moderate	Moderate	High	Moderate	Moderate

## Discussion

The high incidence of DFU, challenges in wound healing, frequent ulcer relapse, and increased amputation rates are the primary contributors to disability, hospitalization, and mortality among individuals with diabetes. The scientific basis underpinning the utilization of PRP is the deficiency of growth factors in chronic wounds. PRP aims to address biological factors that impede the healing process by providing a physiologic pool of cytokines that possess therapeutic efficacy [40]. Au-PRP possesses distinctive biological benefits in the facilitation of wound healing, albeit its precise mechanism remains incompletely elucidated. The potential mechanisms for enhancing wound healing are commonly accepted to be: (1) platelet-rich plasma (PRP) is rich in growth factors that are analogous to those found in the human body. These include transforming growth factor (TGF- β), platelet-derived growth factor (PDGF), keratinocyte growth factor (KGF), Hepatocyte growth factor (HGF), vascular endothelial growth factor (VEGF), fibroblast growth factor (FGF), epidermal growth factor (EGF), and insulin-like growth factor (IGF) [41]. (2) Au-PRP can inhibit excessive inflammatory reaction of wound, regulate the balance of matrix metalloproteinase (MMP) and tissue inhibitor of metalloproteinase (TIMP), and reduce the degradation of wound growth factor and ECM [42]. (3) leukocytes, antimicrobial peptides and platelets in Au-PRP can inhibit the growth of many kinds of bacteria and reduce wound infection [43]. (4) The fibrin present in Au-PRP serves as a scaffold to support various cells involved in the process of wound healing and contributes to wound contraction. In contemporary times, an increasing number of academics hold the belief that Au-PRP exhibits promising potential in the management of DFU owing to its distinctive biological impacts [44].

The rate of healing is a crucial metric for evaluating the efficacy of a medication or intervention on diabetic foot ulcers. The findings of this meta-analysis indicate that the use of Au-PRP can lead to a noteworthy enhancement in the healing rate of DFU in comparison with conventional treatment. The findings are comparable to those of a previous meta-analysis comprising 8 randomized controlled trials and 2 prospective studies, which reported a prevalence of 65.3 vs. 45.5% [45], but it should be noted that certain interventions featured in the meta-analysis comprised of Al-PRP or PDGF. Conversely, the interventions incorporated in this meta-analysis, in accordance with the PICO principle, solely consisted of Au-PRP, thereby rendering the findings more compelling. The ultimate closure of a wound is contingent upon the proliferation of epidermal cells during the wound healing process. In the process of tissue remodeling, various growth factors such as PDGF, KGF, and TGF-β have the potential to stimulate the differentiation of fibroblasts into myofibroblasts. This, in turn, can expedite the contraction of the collagen matrix [46, 47]. Simultaneously, various growth factors such as EGF, IGF, KGF, HGF, among others, have the ability to stimulate the division and proliferation of epithelial cells, thereby expediting the process of wound contraction and re-epithelialization [48, 49]. Moreover, high levels of fibrin present in Au-PRP serve as a scaffold for diverse wound repair cells and facilitate wound contraction. The findings of this meta-analysis indicate that the use of Au-PRP can expedite the pace of wound reduction in comparison with conventional treatment, with a statistically significant difference (*P* < 0.0001). The statistical analysis revealed a significant reduction in ulcer healing time (*P* < 0.00001) concomitant with an increase in the rate of ulcer healing. Patients with DFU are at a significantly elevated risk of amputation, which represents a major contributing factor to disability in this population [6]. The findings of this meta-analysis indicate that the application of Au-PRP can lead to a statistically significant reduction in the amputation rate among patients with DFU when compared to conventional treatment. The presence of multiple active constituents in PRP speeds up wound healing, thereby diminishing the likelihood of wound infection or the propagation of infectious agents. Simultaneously, PRP presents a noteworthy benefit in comparison with traditional therapy by enhancing the healing process of chronic refractory DFU, thereby diminishing the possibility of amputation in DFU patients.

The preparation of Au-PRP is derived from the patient's peripheral venous blood, thereby minimizing the risk of immune-mediated rejection. The procedure requires a relatively small amount of patient blood (approximately 30–50 ml per instance) and can be performed within the ward, which contributes to a less invasive process overall, reducing potential patient discomfort and stress. However, the variability in sample volume and preparation protocols utilized in the included studies may impact the reproducibility and comparability of the results. Therefore, we recommend adopting a universal standardized conventional preparation method, such as the one proposed by Muthu et al. [50]. This method does not require the expense of commercial kits, making it more accessible to a wider range of clinical settings. Several studies [20, 37] have investigated the impact of treatment on hemoglobin, platelet, and coagulation function by conducting re-examinations. The findings indicate that the indices did not exhibit any statistically significant alterations in comparison with their pretreatment levels and did not have any detrimental impact on the patients' blood. The present meta-analysis indicates that there was no statistically significant disparity in the occurrence of unfavorable reactions related to diabetic foot ulcer between the two cohorts. Thus, it is imperative to adhere to the indications and contraindications of Au-PRP during the treatment process to avoid any potential systemic or wound-related adverse reactions. This method of DFU treatment is considered safe.

Our study has several limitations: Initially, it should be noted that certain literatures included in the analysis may exhibit suboptimal quality, as their experimental design may lack rigor. This may potentially compromise the persuasiveness of certain meta-analysis outcomes. The cost of treatment is a crucial consideration for patients with DFU when selecting a treatment option. However, it is noteworthy that only a single study in this research has presented a comparison of treatment costs between the two groups, which precludes a quantitative analysis. Ultimately, the aggregate quantity of investigations and subjects was limited, indicating that more expansive cohort studies are requisite to furnish more precise data.

## Conclusions

The findings of this systematic review and meta-analysis indicate that the use of Au-PRP therapy is a viable and secure therapeutic approach for DFU, as it effectively enhances wound healing. Therefore, it can be concluded that Au-PRP is a viable biological adjuvant therapy option for addressing non-healing DFU.

## Supplementary Information

**Additional file 1**. Medline and EMBASE Search Strategies.

## Data Availability

The datasets used and/or analyzed during the present study are available from the corresponding author on reasonable request.

## References

[1] Harreiter J, Roden M (2019). Diabetes mellitus-Definition, classification, diagnosis, screening and prevention (Update 2019). Wien Klin Wochenschr.

[2] Schmidt AM (2018). Highlighting diabetes mellitus: the epidemic continues. Arterioscler Thromb Vasc Biol.

[3] Zhang P, Lu J, Jing Y, Tang S, Zhu D, Bi Y (2017). Global epidemiology of diabetic foot ulceration: a systematic review and meta-analysis (†). Ann Med.

[4] Armstrong DG, Boulton AJM, Bus SA (2017). Diabetic foot ulcers and their recurrence. N Engl J Med.

[5] Aicale R, Cipollaro L, Esposito S, Maffulli N (2020). An evidence based narrative review on treatment of diabetic foot osteomyelitis. Surgeon.

[6] Hicks CW, Selvarajah S, Mathioudakis N, Sherman RE, Hines KF, Black JH (2016). Burden of infected diabetic foot ulcers on hospital admissions and costs. Ann Vasc Surg.

[7] Ahluwalia R, Lázaro-Martínez JL, Reichert I, Maffulli N (2021). Advances in pharmacotherapy for diabetic foot osteomyelitis. Expert Opin Pharmacother.

[8] Armstrong DG, Swerdlow MA, Armstrong AA, Conte MS, Padula WV, Bus SA (2020). Five year mortality and direct costs of care for people with diabetic foot complications are comparable to cancer. J Foot Ankle Res.

[9] Margolis DJ, Allen-Taylor L, Hoffstad O, Berlin JA (2005). Healing diabetic neuropathic foot ulcers: are we getting better?. Diabet Med.

[10] Eleftheriadou N (2019). Advancing pharmacotherapy for diabetic foot ulcers. Expert Opin Pharmacotherapy..

[11] Ahluwalia R, Maffulli N, Lázaro-Martínez JL, Kirketerp-Møller K, Reichert I (2021). Diabetic foot off loading and ulcer remission: Exploring surgical off-loading. Surgeon.

[12] Pachito DV, Latorraca C, Riera R. Efficacy of platelet‐rich plasma for non‐transfusion use: Overview of systematic reviews. Int J Clin Pract. 2019;73(11). 10.1111/ijcp.13402 31408240

[13] Martinez-Zapata MJ, Martí-Carvajal A, Solà I, Expósito J, Zaror C. Autologous platelet-rich plasma for treating chronic wounds. Cochrane Database Syst Rev (Online). 2016;5(5):CD006899. 10.1002/14651858.CD006899.pub3 PMC9308064 27223580

[14] Meningaud, Jean-Paul, Hersant, Barbara, Picard, Frederic, et al. The growing evidence for the use of platelet-rich plasma on diabetic chronic wounds: A review and a proposal for a new standard care. Wound Repair & Regeneration Official Publication of the Wound Healing Society the European Tissue Repair Society. 2015. 10.1111/wrr.12317 26019054

[15] Page MJ, McKenzie JE, Bossuyt PM, Boutron I, Hoffmann TC, Mulrow CD (2021). The PRISMA 2020 statement: an updated guideline for reporting systematic reviews. BMJ.

[16] Higgins JP, Altman DG, Gøtzsche PC, Jüni P, Moher D, Oxman AD, et al. The Cochrane Collaboration's tool for assessing risk of bias in randomised trials. BMJ. 2011;343:d5928. 10.1136/bmj.d5928. PubMed PMID: 22008217; PubMed Central PMCID: PMCPMC3196245 at www.icmje.org/coi_disclosure.pdf (available on request from the corresponding author) and declare support from the Cochrane Collaboration for the development and evaluation of the tool described; they have no financial relationships with any organisations that might have an interest in the submitted work in the previous three years and no other relationships or activities that could appear to have influenced the submitted work.

[17] Guyatt GH, Oxman AD, Vist GE, Kunz R, Falck-Ytter Y, Alonso-Coello P, et al. GRADE: an emerging consensus on rating quality of evidence and strength of recommendations. Bmj. 2008;336(7650):924–6. 10.1136/bmj.39489.470347.AD. PubMed PMID: 18436948; PubMed Central PMCID: PMCPMC2335261 GRADE’s success has a positive influence on their academic career. Authors listed in the byline have received travel reimbursement and honorariums for presentations that included a review of GRADE’s approach to rating quality of evidence and grading recommendations. GHG acts as a consultant to UpToDate; his work includes helping UpToDate in their use of GRADE. HJS is documents editor and methodologist for the American Thoracic Society; one of his roles in these positions is helping implement the use of GRADE. He is supported by “The human factor, mobility and Marie Curie actions scientist reintegration European Commission grant: IGR 42192—GRADE.” 10.1136/bmj.39489.470347.AD PMC2335261 18436948

[18] Steed DL, Goslen JB, Holloway GA, Malone JM, Bunt TJ, Webster MW (1992). Randomized prospective double-blind trial in healing chronic diabetic foot ulcers. CT-102 activated platelet supernatant, topical versus placebo. Diabetes Care.

[19] Steed DL, Edington HD, Webster MW (1996). Recurrence rate of diabetic neurotrophic foot ulcers healed using topical application of growth factors released from platelets. Wound Repair Regen.

[20] Driver VR, Hanft J, Fylling CP, Beriou JM. A prospective, randomized, controlled trial of autologous platelet-rich plasma gel for the treatment of diabetic foot ulcers. Ostomy Wound Manage. 2006;52(6):68–70, 2, 4 passim. 16799184

[21] Kakagia DD, Kazakos KJ, Xarchas KC, Karanikas M, Georgiadis GS, Tripsiannis G (2007). Synergistic action of protease-modulating matrix and autologous growth factors in healing of diabetic foot ulcers. A prospective randomized trial. J Diabetes Complications.

[22] Jeong SH, Han SK, Kim WK (2010). Treatment of diabetic foot ulcers using a blood bank platelet concentrate. Plast Reconstr Surg.

[23] Setta HS, Elshahat A, Elsherbiny K, Massoud K, Safe I (2011). Platelet-rich plasma versus platelet-poor plasma in the management of chronic diabetic foot ulcers: a comparative study. Int Wound J.

[24] Li L, Wang C, Wang Y, He LP, Yang YZ, Chen LH (2012). Impact of topical application of autologous platelet-rich gel on medical expenditure and length of stay in hospitals in diabetic patients with refractory cutaneous ulcers. Sichuan Da Xue Xue Bao Yi Xue Ban.

[25] Karimi R, Afshar M, Salimian M, Sharif A, Hidariyan M. The effect of platelet rich plasma dressing on healing diabetic foot ulcers. 2016.

[26] Ahmed M, Reffat SA, Hassan A, Eskander F (2017). Platelet-rich plasma for the treatment of clean diabetic foot ulcers. Ann Vasc Surg.

[27] Rainys D, Cepas A, Dambrauskaite K, Nedzelskiene I, Rimdeika R (2019). Effectiveness of autologous platelet-rich plasma gel in the treatment of hard-to-heal leg ulcers: a randomised control trial. J Wound Care.

[28] Elsaid A, El-Said M, Emile S, Youssef M, Khafagy W, Elshobaky A (2020). Randomized controlled trial on autologous platelet-rich plasma versus saline dressing in treatment of non-healing diabetic foot ulcers. World J Surg.

[29] Liao X, Liang JX, Li SH, Huang S, Yan JX, Xiao LL (2020). Allogeneic platelet-rich plasma therapy as an effective and safe adjuvant method for chronic wounds. J Surg Res.

[30] Habeeb T, AA E, H M. Platelet-rich plasma (PRP) bio-stimulant gel dressing in treating chronic non- healing leg and foot ulcers; cost and effectiveness. Randomized Controlled Clinical Trial. 2021.

[31] Hossam EM, Alserr A, Antonopoulos CN, Zaki A, Eldaly W. Autologous platelet rich plasma promotes the healing of non-ischemic diabetic foot ulcers. A randomized controlled trial. 2021. 10.1016/j.avsg.2021.10.061 34896242

[32] Malekpour Alamdari N, Shafiee A, Mirmohseni A, Besharat S (2021). Evaluation of the efficacy of platelet-rich plasma on healing of clean diabetic foot ulcers: a randomized clinical trial in Tehran. Iran Diabetes Metab Syndr.

[33] Helmy Y, Farouk N, Ali Dahy A, Abu-Elsoud A, Fouad Khattab R, Elshahat Mohammed S (2021). Objective assessment of platelet-rich plasma (PRP) potentiality in the treatment of chronic leg ulcer: RCT on 80 patients with venous ulcer. J Cosmet Dermatol.

[34] Meamar R, Ghasemi-Mobarakeh L, Norouzi MR, Siavash M, Hamblin MR, Fesharaki M (2021). Improved wound healing of diabetic foot ulcers using human placenta-derived mesenchymal stem cells in gelatin electrospun nanofibrous scaffolds plus a platelet-rich plasma gel: a randomized clinical trial. Int Immunopharmacol.

[35] Mohammadi Tofigh A, Tajik M (2022). Comparing the standard surgical dressing with dehydrated amnion and platelet-derived growth factor dressings in the healing rate of diabetic foot ulcer: a randomized clinical trial. Diabetes Res Clin Pract.

[36] Singh SP, Kumar V, Pandey A, Pandey P, Gupta V, Verma R (2018). Role of platelet-rich plasma in healing diabetic foot ulcers: a prospective study. J Wound Care.

[37] Li L, Chen D, Wang C, Yuan N, Wang Y, He L (2015). Autologous platelet-rich gel for treatment of diabetic chronic refractory cutaneous ulcers: A prospective, randomized clinical trial. Wound Repair Regen.

[38] G F, M H, WA S. The use of autologous platelet concentrate acti-vated by autologous thrombin (APC+) is effective and safe in the treatment ofchronic diabetic foot ulcers: a randomized controlled trial. Fifth International Symposium Diabetic Foot Noordwijkerhout The Netherlands:2007.

[39] Saldalamacchia G, Lapice E, Cuomo V, Feo ED, Vaccaro O (2004). A controlled study of the use of autologous platelet gel for the treatment of diabetic foot ulcers. Nutr Metab Cardiovasc Dis.

[40] Martí-Carvajal A, Gluud C, Nicola S, Simancas-Racines D, Reveiz L, Oliva P (2015). Growth factors for treating diabetic foot ulcers. Cochrane Database Syst Rev.

[41] Everts P, Onishi K, Jayaram P, Fábio J, Mautner K (2020). Platelet-rich plasma: new performance understandings and therapeutic considerations in 2020. Int J Mol Sci.

[42] Li T, Ma Y, Wang M, Wang T, Wei J, Ren R, et al. Platelet-rich plasma plays an antibacterial, anti-inflammatory and cell proliferation-promoting role in an in vitro model for diabetic infected wounds. Infect Drug Resist. 2019;12. 10.2147/IDR.S186651 PMC6357877 30774397

[43] Mariani E, Filardo G, Canella V, Berlingeri A, Bielli A, Cattini L, et al. Platelet-rich plasma affects bacterial growth in vitro. Cytotherapy. 2014. 10.1016/j.jcyt.2014.06.003 25108654

[44] Shao S, Pan R, Chen Y (2020). Autologous platelet-rich plasma for diabetic foot ulcer. Trends Endocrinol Metab.

[45] Tasmania, Del, Pino-Sede?o, María, Trujillo-Martín, Isabel, et al. Platelet-rich plasma for the treatment of diabetic foot ulcers: A meta-analysis. Wound repair and regeneration : official publication of the Wound Healing Society and the European Tissue Repair Society. 2018. 10.1111/wrr.12690 30575212

[46] Badiu D, Vasile M, Teren O. Regulation of wound healing by growth factors and cytokines. 2011.

[47] Gardner JC, Wu H, Noel JG, Ramser BJ, Pitstick L, Saito A, et al. Keratinocyte growth factor supports pulmonary innate immune defense through maintenance of alveolar antimicrobial protein levels and macrophage function. Am J Physiol Lung Cell Mol Physiol. 2016;310(9):ajplung.00363.2015. 10.1152/ajplung.00363.2015 PMC4867350 26919897

[48] Kim YS, Lew DH, Tark KC, Rah DK, Hong JP (2010). Effect of recombinant human epidermal growth factor against cutaneous scar formation in murine full-thickness wound healing. J Korean Med Sci.

[49] Chicharro-Alcántara D, Rubio-Zaragoza M, Damiá-Giménez E, Carrillo-Poveda JM, Cuervo-Serrato B, Peláez-Gorrea P (2018). Platelet Rich plasma: new insights for cutaneous wound healing management. J Funct Biomater..

[50] Muthu S, Krishnan A, Ramanathan KR. Standardization and validation of a conventional high yield platelet-rich plasma preparation protocol. Ann Med Surg (Lond). 2022;82:104593. 10.1016/j.amsu.2022.104593. 10.1016/j.amsu.2022.104593 PMC9577528 36268335

