# Supplementary material for: Efficacy and safety of autologous platelet-rich plasma for diabetic foot ulcer healing: a systematic review and meta-analysis of randomized controlled trials
Source: J Orthop Surg Res. 2023 May 19;18:370. doi: 10.1186/s13018-023-03854-x (PMC10197861; doi:10.1186/s13018-023-03854-x)
Supplement: Supplementary file 1 — Additional file 1. Medline and EMBASE Search Strategies. [file 13018_2023_3854_MOESM1_ESM.docx]

Medline search strategy:

((((((("Platelet Rich Plasma"[MeSH Terms]) OR "Plasma, Platelet-Rich"[MeSH Terms]) OR "Platelet-rich Plasma Gel"[MeSH Terms]) OR "PRP"[MeSH Terms]) OR "Platelet-Rich Plasma"[MeSH Terms])) AND (("Diabetic Foot"[MeSH Terms]) OR (((((("Foot Ulcer, Diabetic"[MeSH Terms]) OR "Feet, Diabetic"[MeSH Terms]) OR "Diabetic foot ulcer wounds"[MeSH Terms]) OR "Diabetic Feet"[MeSH Terms]) OR "Diabetic foot ulcer"[MeSH Terms]) AND "Diabetic foot Wound"[MeSH Terms]))) AND (("Randomized Controlled Trial"[Publication Type]) OR (("Randomized"[MeSH Terms]) OR "Placebo"[MeSH Terms])).

EMBASE search strategy:

((((((('Platelet Rich Plasma'/exp) OR 'Plasma, Platelet-Rich'/exp) OR 'Platelet-rich Plasma Gel'/exp) OR 'PRP'/exp) OR 'Platelet-Rich Plasma'/exp)) AND (('Diabetic Foot'/exp) OR (((((('Foot Ulcer, Diabetic'/exp) OR 'Feet, Diabetic'/exp) OR 'diabetic foot ulcer wounds'/exp) OR 'Diabetic Feet'/exp) OR 'Diabetic foot ulcer'/exp) AND 'Diabetic foot Wound'/exp))) AND (('Randomized Controlled Trial'/exp) OR (('Randomized'/exp) OR 'Placebo'/exp)).
